# The Extent, Nature and Environmental Health Implications of Cottage Industries in Johannesburg, South Africa

**DOI:** 10.3390/ijerph120201894

**Published:** 2015-02-05

**Authors:** June Teare, Tahira Kootbodien, Nisha Naicker, Angela Mathee

**Affiliations:** 1 South African Medical Research Council, P.O. Box 19070, Tygerberg 7505, South Africa; E-Mails: tahira.kootbodien@mrc.ac.za (T.K.); nisha.naicker@mrc.ac.za (N.N.); angie.mathee@mrc.ac.za (A.M.); 2 School of Public Health, Faculty of Health Sciences, University of the Witwatersrand, 1 Jan Smuts Avenue, Braamfontein, Johannesburg 2000, South Africa

**Keywords:** cottage industry, toxic metals, lead, mercury, cadmium, arsenic

## Abstract

Cottage industries comprise a sub-group of informal sector income generation activities which are conducted in the home environment and organized around families or households. Cottage industry workers may be at risk of exposure to harmful substances associated with their work, and given the lack of separation of cottage industry activities from living spaces, their families and neighbors may similarly be at risk of exposure. This study was undertaken to determine the extent and nature of cottage industries in five neighborhoods in Johannesburg (South Africa) A cross-sectional survey was conducted across five communities in Johannesburg in 2012. Data on metal-related cottage industry activities were collected through the administration of a pre-structured questionnaire. Metal-related cottage industry activities were defined as car repairs, spray painting, scrap metal recycling, electrical appliance repairs, welding, hairdressing and metal jewelry making. One fifth of the households interviewed were operating one or more cottage industries associated with the use of toxic substances. Therefore, the potential exists for associated ill health effects in a considerable proportion of the population. Further research is needed to fully assess exposure to the harmful aspects of cottage industry, as are scaled up campaigns to increase awareness of the risks and correct handling of toxic substances.

## 1. Introduction

Cottage industries comprise a sub-group of income generation activities under the umbrella of the informal sector, and are characterized by their conduct in the home setting, and their organization around families or households [1]. Home-based work is an important source of employment for many, especially in developing countries [2], and is widely regarded as a strategy for providing employment and alleviating or surviving poverty [3].

However, a wide range of pollutants may be used in cottage industries. Exposure to harmful substances in cottage industries may be exacerbated by limited knowledge and awareness of the associations between cottage industry processes and ill health outcomes [1]. Furthermore, given the lack of separation of cottage industry activities from living spaces, families and other household members may similarly be at risk of exposure to a range of harmful substances [4]. In this regard, those who spend a major portion of their time in the home environment may be at particular risk, for example, young children (especially those with pica or pronounced hand-to-mouth behavior), those with pre-existing ill-health conditions, the elderly, and pregnant women. Cottage industries are almost completely unregulated and exempt from worker compensation laws and other occupational health and safety regulations [1]. However, certain cottage industries involve the use of highly toxic metals such as lead [5], mercury, cadmium and arsenic, which have been associated with a range of detrimental effects on health, including reductions in intelligence quotient scores, hyperactivity, shortened concentration spans, and aggressive or violent behavior [6,7]. Table 1 shows the toxic metal, the associated cottage industry relevant to this paper and the concomitant health effect.

**Table 1:** Toxic metals and concomitant health effects associated with type of cottage industry.

Toxic Metal	Cottage Industry	Health Effect
**Lead**	Electrical appliance repair [8,9] Hairdressing [10] Car repairs [11,12,13] Welding [11] Spray painting [14,15] Scrap metal recycling [8,16] Metal jewelry making [17]	Ill health effects include headache, irritability, abdominal pain, fatigue, nausea, vomiting, problems with intellectual development, convulsions, coma, renal failure, death, hypertension, cognitive impairment, tremor of the hands, excitability, memory loss, insomnia [18,19], cardiac conduction disturbances [20]. Children in particular are susceptible to the ill health effects of lead exposure due to their permeable blood-brain barrier and high gastrointestinal uptake. Children may be affected by behavioural disturbances, learning and concentration difficulties [19].
**Mercury**	Electrical appliance repair [8,9]	Injury to the lungs and the neurological system, anxiety [18,19].
**Arsenic**	Electrical appliance repair [8,9]	Skin hyperpigmentation, skin cancer, cancers of the liver, lungs and bladder, diabetes, blood vessel damage, and peripheral nerve damage (presenting as numbness or tingling in hands and feet) [18,19,21].
**Cadmium**	Electrical appliance repair [8,9] Welding [22] Spray painting [14] Metal jewelry making [17]	Chronic lung disease [8], kidney dysfunction [23] and osteoporosis [24].

The aim of this study is to describe the extent and nature of cottage industries in Johannesburg, South Africa.

## 2. Methods

### 2.1. Data Collection

The Health, Environment and Development (HEAD) study is a Johannesburg-based, household level study, which was initiated in 2006. Annual cross-sectional surveys are conducted in five lower socio-economic communities for the collection of data on socio-demographic factors, as well as living conditions and health status [25]. The entire HEAD study is designed in this way to assess trends in environmental conditions and health status of impoverished communities. The HEAD study is undertaken in Riverlea, an apartheid era township; Bertrams, an old, degraded suburb of Johannesburg, with a protracted history of displacement and migration; Hillbrow, a densely populated, high rise, inner city area; Braamfischerville, a democratic era low-cost, mass-based housing development; and Hospital Hill, an informal settlement situated to the west of Johannesburg. These five study sites characterize the main housing options available to the urban poor living in Johannesburg.

Households were randomly selected using town planning maps and/or aerial photographs; vacant and non-residential buildings were excluded. In Bertrams, Riverlea and Braamfischerville dwellings for inclusion were randomly selected at the start of the study. In the informal settlement of Hospital Hill a convenience sample of dwellings was taken, while in the high-rise neighborhood of Hillbrow apartments were systematically sampled (random sampling of apartment buildings, followed by floors and then individual apartments).

For this analysis data on socio-economic status and metal-related cottage industry activities in the five HEAD study neighborhoods (Riverlea, Bertrams, Hillbrow, Braamfischerville and Hospital Hill) were extracted for the year 2012. The question posed was, “Does anyone regularly do any of the following at home to make money?” The cottage industry categories presented were: fix cars, spray painting of cars, make metal jewelry, welding, fix electrical appliances, scrap metal recycling, hairdressing, and other, where respondents were required to state the type of cottage industry. Response options were “Yes”, “No”, “Don’t know” and “Refused to answer”.

### 2.2. Procedure

Each participant gave written informed consent prior to commencement of the study. Ethical approval for this study was granted by the Ethics Committee of the University of Witwatersrand, South Africa (Protocol number M10471). A total of 548 interviews were conducted in the five study areas. All participants gave their informed consent for inclusion in the study.

The field work took place between August and September 2012. An adult, over the age of 18 years, in each household was randomly selected to be interviewed. Pre-structured questionnaires were administered by environmental health students from the University of Johannesburg, who had been trained in interviewing techniques. Questions were orally translated for participants who did not speak English. Persons who were not immediately available (owing to circumstances such as work or hospitalisation) were contacted the following month over the weekend. The overall response rate was 68% (548/805), with 12% refusals (96/805) and 20% (161/805) participants not available.

### 2.3. Data Analysis

Point estimate prevalence of cottage industries was presented as percentages and 95% confidence intervals (CI). Difference in prevalences was described using chi-square test. All analyses were conducted using Stata Statistical Software: Release 12 (StataCorp LP., College Station, TX, USA, 2011).

## 3. Results

### Prevalence and Types of Cottage Industries

**Figure 1:**
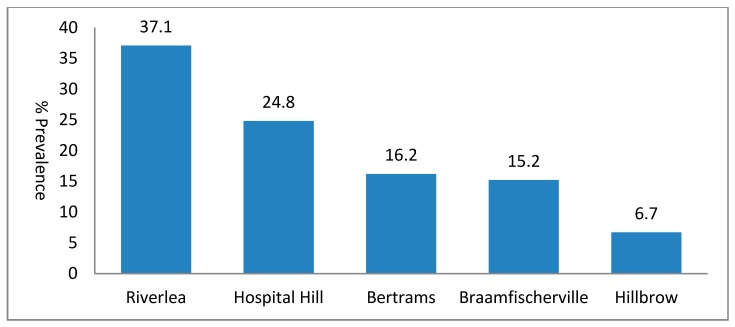
Overall prevalence of cottage industry in the five study sites.

One hundred and five (19.2%, 95% CI: 16.1%–22.6%) out of a total sample of 548 households reported the operation of at least one metal-related cottage industry within their homes. Of these, 32.4% were operating multiple metal-related cottage industries. For example, in Riverlea, a household would commonly operate electrical appliance repairs and car repairs. The prevalence of cottage industries varied significantly from one neighborhood to another (chi-square test, *p* < 0.001). As shown in Figure 1, Riverlea (37.1%; 95% CI: 23.8%–40.2%) and Hospital Hill (24.8%; 95% CI 14.9%–34.4%) had the highest prevalence of cottage industries. In Hillbrow, on the other hand, only 6.7% (95% CI 5.1%–17.1%) of households operated a cottage industry, possibly due to the nature of the neighborhood – Hillbrow is a densely populated high-rise neighborhood with limited space for cottage industries. Cottage industry prevalence in Bertrams and Braamfischerville was 16.2% and 15.2%, respectively.

Overall, the most widespread types of cottage industry were electrical appliance repairs (25.8%) and hairdressing (25.8%).

## 4. Discussion

This study highlights the scale of operation of cottage industries in settings of relative poverty in Johannesburg—19% in the total study, and up to 37% of households in particular neighborhoods. The most common cottage industries were electrical repairs (25.8% in the total sample and up to 53.9% in Riverlea) and hairdressing (25.8% in the total sample and up to 57.7% in Hospital Hill).

Both electrical appliance repairs and hairdressing are associated with the use of hazardous substances. For example, repair work on electrical appliances may result in exposure to arsenic used in microwave circuits and light-emitting diodes [18]; cadmium in cathode-ray tube (CRT) screens such as those used in television sets and old computer monitors; lead in the glass of CRT screens, batteries, solder, and cable sheathing [18]; and mercury in fluorescent lamps, thermostats, and relay switches used to open and close electrical circuits [9]. Those involved in hairdressing are at risk of exposure to hundreds of harmful chemicals [26] and volatile organic compounds such as lead acetate [10]. Lead acetate, found in certain hair-dyes, is water soluble and easily transferred to hands and other surfaces, such as counter tops and hairdressing equipment, with the potential for hand-to-mouth transfer of lead in adults as well as children [10]. Children are particularly vulnerable to the neurotoxic effects of lead [6,7,18,19]. Even low-level lead exposure in children results in impaired cognitive function which presents as a loss in intelligence quotient [27]. Interestingly, informal car lead battery recycling, which is well-documented in many developing countries [28,29,30,31,32], was not reported by respondents interviewed in this study.

Amongst other human activities such as mining and the use of unleaded petrol, cottage industry has been shown to be a source of exposure to lead [33]. The uncontrolled use of hazardous substances in cottage industries poses a health threat to cottage industry workers themselves, as well as to their families and neighbors. The public health impact is therefore potentially significant. Protecting the health of people involved in home-based enterprises is recognized to be a major challenge, especially given the informal nature and relative concealment of cottage industries within dwellings and backyards. Cottage industry involvement in the HEAD study undertaken in 2006 showed an overall prevalence of 14% compared to the present study (19%), with a 4% involvement in more than one cottage industry activity [34]; present study results show a marked increase (32.4%) for households involved in multiple cottage industries. Given the scale of operation of cottage industries, which may increase further in the light of the stagnating formal economy in South Africa, public health action is warranted. At the very least, and in the short term, awareness programs on relevant hazards, and the promotion of protection measures such as good ventilation in the workplace, the use of protective clothing, and measures to reduce contamination in the home environment, are of particular importance to protect workers, their families and neighbors, and especially vulnerable groups such as young children.

A limitation of this study is that actual exposure to metal toxicity was not assessed, neither was the presence or absence of personal protection equipment. Further work is needed to assess the occupational metal exposure associated with selected cottage industry activities and the impact on the communities’ health.

## 5. Conclusions

Further research is needed to fully assess the health implications of exposure of cottage industry workers, their families and neighbors, to the harmful substances associated with their occupations. The contribution of cottage industries to household exposure needs to be characterized in relation to other known local sources of exposure to hazardous substances, for example, from industrial processing plants and mining activities [35]. In light of the evidence from this study of widespread prevalence of cottage industries associated with hazardous substances, there is a clear need for scaled-up health action. Apart from immediate strategies and action to reduce exposure, the roles of the occupational and environmental health sectors need to be explored in relation to public health protection.
